# The Diagnosis and Prognosis of Acute Pneumonia—II

**Published:** 1910-08-27

**Authors:** J. Walter Carr

**Affiliations:** Physician to the Royal Free Hospital.


### Hospital Clinics.

### THE DIAGNOSIS AND PROGNOSIS OF ACUTE PNEUMONIA—II.

By J. WALTER CARR, M.D., F.R.C.P., F.R.C.S., Physician to the Royal Free Hospital.

A Lecture delivered at the Polyclinic, Chenies Street, W.C., on Thursday, July 14, 1910,

Now let us turn to difficulties in the diagnosis of pneumonia which arise, not from symptoms, but in connection with physical signs, these being more or less absent or, if present, misleading in their character. We must recognise the no inconsiderable difficulty of examining these patients. You are called hurredly to a man who has just had a severe rigor. You find him buried in the depths of a large feather bed, covered with a couple of sheets, three or four blankets, and an eiderdown quilt, and it is difficult, in face of the protests of his relations, to disinter him from these numerous coverings, and actually to reach his skin, especially if, as is probable at this stage, there is no trained nurse present. As the patient has just had one rigor, the friends and relations consider that the slightest exposure will bring on another. Consequently they strongly resent a thorough physical examination, as does also the patient. At most you may only be able to get him on to his side in order to examine the back, and if the physical signs are but slightly different on the two sides it is difficult to make a satisfactory examination with the patient lying in this position. Moreover, at this time, even if you can make a thorough examination, it is most probable you will not detect any definite abnormality. When you want to make a further examination on the second or third day, or later, when the patient is obviously weaker, his friends and relatives are still more resentful, and consider that if you were not skilful enough to discover the cause at your first examination you will not be able to find it later on.

### Delayed Pneumonia.

Passing from these difficulties, which are real enough in practice, we have to recognise that in a considerable number of cases there is a remarkable delay in the development of the physical signs, more especially in the pneumonias of children. Two three, four, sometimes five days may pass, and yet a most exhaustive investigation fails to reveal any sign of consolidation, although you may be convinced from the character of the symptoms that the patient is suffering from acute pneumonia. I am quite sure that in some cases a child passes through an attack, the crisis comes and the patient gets well, and no physical signs are ever detectable, especially if the illness lasts only a few days. I do not know why this is so; some people have suggested that there may be a pneumonic infection of the blood without local manifestation in the lungs. On the other hand, we have the old explanation of the so-callel central pneumonia, in which the consolidation never reaches the surface. I am loth to part with this explanation, it is so invaluable in these otherwise inexplicable cases; I should feel it almost as great a loss as to part with dentition as a cause of disease in infancy, with worms as an explanation of childish ailments, or with suppressed gout for symptoms met with in later life! But I have not met with anybody who has ever seen central pneumonia in the post-mortem room. Supposing, however, that physical signs do develop, remember they may be by no means those described in the text-books. There is impairment of resonance, but certainly not absolute dulness, especially in the early stages of the disease. Then on auscultation we expect loud bronchial or tubular breathing of the most unmistakable character. What we frequently find, especially at first, is simply weak breathing. That is often the earliest auscultatory sign of pneumonia in a child. We hope to meet with the characteristic sharp pneumonic crepitation, but often it is a very transitory phenomenon.

### Pleuro-Pneumonia and Empyema.

Again, the physical signs may be marked enough, but they are those not of consolidation, but of fluid, absolute dulness, absent breath sounds, loss of vocal fremitus, loss of vocal resonance. The reason is obvious enough. We are dealing not with pneumonia but with pleuro-pneumonia. It has given rise to effusion, and through this the signs of the lung consolidation are not detectable. Fortunately in these cases we can generally recognise the pneumonia by the characteristic symptoms, and the pleural effusion by the physical signs. Later on there is often another still more serious danger, and that is missing an empyema. Everybody knows that acute pneumonia is very liable to be followed by empyema, but it is extraordinary how in actual practice we are apt to become obsessed with the idea that we have still to deal with lung consolidation, and so fail to recognise indications of the development of fluid. One reason, perhaps, is that we cling too closely to the old text-book descriptions of an internal abscess. We expect to meet with a markedly hectic type of temperature: a rise to 104° every night, and a drop, with profuse sweating, to 100° or lower in the morning, whereas in a large number of cases of pneumococcal empyema we do not see anything of the sort; we have merely slight irregular rises of temperature, and because the fever is so slight, much lower than it was throughout the course of the acute pneumonia, we talk about chronic pneumonia or unresolved pneumonia, conditions which are exceedingly rare. Again, we associate with empyema the idea not only of marked dulness, but also of absent breath sounds and loss of vocal resonance, and we fail to recognise that in a large number of these cases we have bronchial, tubular, or even cavernous breathing of the most marked type, over pus. In fact, I regard auscultation as frequently misleading rather than helpful, especially in the empyemas of childhood. The essential features are marked dulness, the sort of dulness which you can detect without hearing a sound, merely by the increased resistance, with obvious illness. The patient does not improve. If you like to have a blood count made you will probably find a high leucocytosis, but this does not help very much, because there is high leucocytosis during and for a short time after an attack of pneumonia. When a patient after acute pneumonia has some irregular fever, however slight, persistent malaise and absolute dulness at one base, it does not matter in the least what the auscultatory signs are: the duty of the attendant practitioner is plain, and that is to explore with a needle. The quantity of pus is sometimes very small, therefore it is most important in making your exploration to pick out the centre of the dull area into which to put the needle, otherwise it is easy to miss the pus. Do not go near the edge of the dulness.

Another mistake, less serious, but sometimes made, is in connection with apical pneumonia. It is an old clinical aphorism that in dealing with acute apical consolidation we should never come to an immediate diagnosis. Remember that whilst an acute consolidation may be pneumonic, it may also possibly be tuberculous in its nature. It is not often the mistake is made of diagnosing acute tuberculous consolidation—what used to be known as caseous pneumonia—as a true acute pneumonia. Much more frequently the converse error is made of diagnosing acute pneumonia as acute tuberculosis. A boy is brought up to the hospital; the mother tells you he has been ill more or less for several weeks. Afterwards, perhaps, when circumstances lead you to inquire more closely, you find that in the first few weeks of his so-called illness he was scarcely out of health at all; it was only in the last week or ten days that he was ill in bed. He now looks very ill. You examine him and find well-marked signs at one apex, which suggest that his lung is rapidly breaking down. You tell the mother you fear the boy has got acute galloping consumption, that the prognosis is very serious, at any rate he had better be got away as soon as possible to a sanatorium if he is fit to travel. A fortnight later you see him again, and very likely he is quite well, with no difference whatever between the physical signs at the two apices. The whole thing was a preliminary malaise, which for some reason I cannot explain often precedes acute pneumonia, and then a definite attack of that disease, and you saw the boy when he was looking ill as a result of the pneumonia, but when he was really convalescing. The mistake is not serious from the point of view of the patient, but it may be disastrous for the prestige of the practitioner. It is most difficult, sometimes for a time impossible, I think, to distinguish by physical signs between well-marked consolidation of the lung, in which the inflammatory exudation only is beginning to break down, the lung tissue itself remaining intact, and commencing excavation in which the exudation and also the lung itself are breaking down together, as in tuberculous disease. You must not rely on physical signs only for the distinction between these two conditions.

### A Note on Prognosis.

Now a few words on the second part of my subject: prognosis in pneumonia. In pneumonia, as in all acute diseases, prognosis resolves itself into three considerations. The first and most obvious question, of course, is, Will the patient recover? The second, which follows from that, is, If he recovers, how long is he likely to be about it? And the third, which follows from the second, is, Will his recovery, if it occur, be complete and permanent, or will he be left with some weakness or disability, some tendency to recurrence, some probability of shortened life?

First of all with regard to the prospects of recovery. One point which seems very important, and yet is by no means so important as it appears, is the area of lung affected. At first you would say the greater the area of lung involved the worse the prognosis. In some measure, this is true; double pneumonia is more serious than when the disease involves only one lung, and pneumonia which involves a whole lung is more serious than that which affects only a part. Yet it is remarkable how comparatively little influence this has upon prognosis. Some of the most serious cases of pneumonia involve only a comparatively small area of lung, and *vice versâ*.

A second consideration, and a much more important one, is the resistive power of the patient, practically the condition of his heart and vessels and of his nervous system. This depends on several further considerations. In the first place, on the kind of tissues which he inherits. You may be able to estimate this or you may not, but it is an important factor in the prognosis. We talk about a patient's constitution; we cannot define it, we do not know what it means, but we know it is a very real thing in the prognosis of acute disease. Secondly, there is the age of his tissues. Inevitably as a man gets older his vitality gets less and his resistive power diminishes. You may explain this in various ways, but these do not concern us now; we have to deal with the fact. In childhood, except in the earliest months of infancy, the prognosis in acute pneumonia—I am not speaking of broncho-pneumonia—is remarkably favourable. So much so that I am prepared to make the reputation of any remedy you like to mention if I may choose the age of the patients to whom I may administer it. I always profoundly distrust statistics as to the effects of remedies upon pneumonia unless the fullest information is given as to the age of the patients. Children do not die of pneumonia unless there are some special complications, or unless they are already weakened or exhausted by some pre-existing disease. There are, of course, exceptions, but that is the general rule. The child may look desperately ill, but will probably recover for all that. In older people the prognosis becomes increasingly unfavourable, and yet even old people may recover most satisfactorily. I have seen several patients over seventy years of age make a complete and perfect convalescence from a well-marked attack. We medical men know, of course, that age is not to be measured as men ordinarily reckon it, by the number of years, but by the condition of the tissues, and particularly of the heart and arteries. It is in what Sir Andrew Clark termed the "spent man" that pneumonia is almost invariably fatal. One man may be "spent" at fifty years of age, another may still be young at seventy-five. In old people there is a marked danger of sudden collapse; they may seem to be going on fairly favourably, and twenty-four hours later may be moribund. Note, too, that sometimes they may go on day after day, and you may think that the crisis is now due and surely will soon come and the patient recover; but only too often it does not come in these elderly people, and the case ends fatally. Why that is I do not know; perhaps they have not the capacity of forming the anti-bodies, or whatever they are, which neutralise the poison in pneumonia and so they succumb. The third factor to be considered in connection with the resistive power is how the patient has used his tissues. Here especially the baneful influence of alcohol manifests itself; the alcoholic patient is one of the worst subjects for pneumonia. Probably he becomes delirious, and the supervention of serious delirium, which keeps him awake and exhausts him, is of the worst prognostic significance. It weakens the heart and nervous system, and so kills the patient.

Lastly, in connection with resistive power, we have to consider the influence of other diseases. Obviously, if a pneumonia patient is already suffering from heart disease or pre-existing lung disease, such as bronchitis or emphysema, or from renal disease, the prognosis is correspondingly serious.

Another factor in the prognosis, one which at present we are utterly unable to estimate, but which is of extreme importance, is the virulence of the infecting germ or its association with other pathogenic microbes. Take, for instance, the pneumonia which so frequently supervenes on influenza. Apparently the combination of the influenza germ with the pneumococcus increases the virulence of the latter, for we know that the prognosis in these cases is much more grave than in uncomplicated ones. Again, we meet from time to time with pneumonia occurring in a young adult; there is no suspicion or evidence of alcoholism, there is no pre-existing disease, the previous health has been good, all the organs are apparently healthy, yet that patient succumbs. We do not know why, we can only attribute it to the virulence of the infecting agent. A year or two ago I saw several times in consultation a young and previously healthy girl, who lived in good surroundings and under favourable conditions. She was suffering from acute pneumonia obviously of a severe type. It went on for eight or ten days, and then came the crisis. We hoped all would be well. Twenty-four hours later she had a second rigor and rapid development of pneumonia in the other lung; this second attack was too much for her, and three or four days later, on the anniversary of her eighteenth birthday, she died. At the outset all seemed favourable, and yet for some reason the patient did badly; evidently the disease was of a malignant type. We know, too, that in so-called epidemic pneumonia, of which there was a notable outbreak some years ago in Middlesbrough, that the prognosis is much more serious than in sporadic cases and the death rate very high. We can only attribute it to increased virulence of the germ, and with the increased virulence increased infectivity. We have no means of estimating this. To some extent we can gauge the virulence from the height of the fever, but not altogether, for in some of the most unfavourable cases of pneumonia the temperature is but little raised throughout. A high temperature indicates a severe attack, but it also indicates good resistive power on the part of the patient and shows that he is making a good fight. So that a prognosis based on temperature must be a very guarded one.

Next as to the duration of the disease. The ordinary teaching, I suppose, is that if the crisis has not come by the tenth day some complication is probably present. I have, however, seen several cases of pneumonia in childhood in which the fever has persisted for three, four, or five weeks; then the crisis has come, with no complication, and the whole thing has cleared up. I have seen the disease start in the apex of one lung, spread down to the base of that lung, then start on the other side, take several weeks over the whole course, and still the patient recover. I show you the charts of a case which I have under my care at the present time, a child two and a half years old, who was admitted on the fourth day of acute pneumonia, and he is still running a temperature. We can trace the course of the consolidation; it commenced at the base of the right lung, spread up to the apex, and now there is an indication of extension to the other lung.* Perhaps these long continued cases are made up of intercurrent relapses; but remember that it is never wise to fix a definite duration for an attack of pneumonia. You may safely tell the friends the chances are that the disease will come to an end before the tenth day, but that it may last much longer than this. When the disease lasts so long in adults the prognosis is very unfavourable; but children usually recover.

Lastly, if the patient survive, will his recovery be complete and permanent? We can answer this question quite definitely. There is the danger of empyema always to be thought of, although even then, if the patient is properly treated, the prognosis is not unfavourable. But apart from that possibility, there is no acute disease which leaves less mischief behind it than does pneumonia; there are few cases in which we have greater difficulties to overcome. The patient may be apparently at the point of death, yet, if once the crisis occur we know that he will recover rapidly and completely, without any disability, either as regards mental impairment, physical vigour, or any probability of shortened life.

* The child has since recovered completely.

